# Features of CRISPR-Cas Regulation Key to Highly Efficient and Temporally-Specific crRNA Production

**DOI:** 10.3389/fmicb.2017.02139

**Published:** 2017-11-03

**Authors:** Andjela Rodic, Bojana Blagojevic, Magdalena Djordjevic, Konstantin Severinov, Marko Djordjevic

**Affiliations:** ^1^Faculty of Biology, Institute of Physiology and Biochemistry, University of Belgrade, Belgrade, Serbia; ^2^Multidisciplinary PhD Program in Biophysics, University of Belgrade, Belgrade, Serbia; ^3^Institute of Physics Belgrade, University of Belgrade, Belgrade, Serbia; ^4^Waksman Institute of Microbiology, Rutgers University, Piscataway, NJ, United States; ^5^Skolkovo Institute of Science and Technology, Skolkovo, Russia

**Keywords:** CRISPR-Cas activation, pre-crRNA processing, CRISPR regulation, crRNA generation, biophysical modeling

## Abstract

Bacterial immune systems, such as CRISPR-Cas or restriction-modification (R-M) systems, affect bacterial pathogenicity and antibiotic resistance by modulating horizontal gene flow. A model system for CRISPR-Cas regulation, the Type I-E system from *Escherichia coli*, is silent under standard laboratory conditions and experimentally observing the dynamics of CRISPR-Cas activation is challenging. Two characteristic features of CRISPR-Cas regulation in *E. coli* are cooperative transcription repression of *cas* gene and CRISPR array promoters, and fast non-specific degradation of full length CRISPR transcripts (pre-crRNA). In this work, we use computational modeling to understand how these features affect the system expression dynamics. Signaling which leads to CRISPR-Cas activation is currently unknown, so to bypass this step, we here propose a conceptual setup for *cas* expression activation, where *cas* genes are put under transcription control typical for a restriction-modification (R-M) system and then introduced into a cell. Known transcription regulation of an R-M system is used as a proxy for currently unknown CRISPR-Cas transcription control, as both systems are characterized by high cooperativity, which is likely related to similar dynamical constraints of their function. We find that the two characteristic CRISPR-Cas control features are responsible for its temporally-specific dynamical response, so that the system makes a steep (switch-like) transition from OFF to ON state with a time-delay controlled by pre-crRNA degradation rate. We furthermore find that cooperative transcription regulation qualitatively leads to a cross-over to a regime where, at higher pre-crRNA processing rates, crRNA generation approaches the limit of an infinitely abrupt system induction. We propose that these dynamical properties are associated with rapid expression of CRISPR-Cas components and efficient protection of bacterial cells against foreign DNA. In terms of synthetic applications, the setup proposed here should allow highly efficient expression of small RNAs in a narrow time interval, with a specified time-delay with respect to the signal onset.

## Introduction

**Figure 1:**
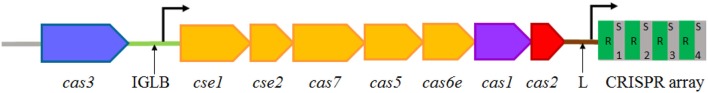
A scheme of a Type I-E CRISPR-Cas system from *E coli* (Al-Attar et al., 2011; Makarova et al., 2011, 2015). The *cas* genes and the CRISPR array are indicated. R and S within the CRISPR array correspond, respectively, to repeats and spacers; note that the spacer sequences differ from each other, and are labeled by consecutive numbers (1, 2, 3…). IGLB and L correspond to intergenic regions where promoters for the Cascade complex genes (*cse1,2, cas7,5,6e*) and the Cas1,2 adaptation proteins (IGLB) and the CRISPR array (L) are located. The two promoters within IGLB and L are indicated by arrows. One of the Cas proteins (Cas6e) is responsible for processing pre-crRNA to crRNA. The effector Cascade complex is composed of proteins encoded by genes marked with yellow color. It binds crRNA, which recognizes invading DNA. Once recognized, foreign DNA is destroyed by the product of *cas3* (Brouns et al., 2008).

CRISPR-Cas are adaptive immune systems, which defend prokaryotic cells against foreign DNA, including viruses and plasmids. A CRISPR-Cas system consists of a CRISPR (Clustered Regularly Interspaced Short Palindromic Repeats) array and associated *cas* genes (Makarova et al., 2006; Barrangou et al., 2007; Brouns et al., 2008; Hille and Charpentier, 2016). CRISPR arrays consist of identical direct repeats (R) of about 30 bp in length, interspaced with spacers (S) of similar length and variable sequence. Spacer sequences are often complementary to fragments of viral or plasmid DNA. A match between a CRISPR spacer and invading phage (bacterial virus) sequence provides immunity to infection (Barrangou et al., 2007; Hille and Charpentier, 2016). The entire CRISPR locus is initially transcribed as a long transcript (called pre-crRNA) (Pougach et al., 2010; Pul et al., 2010), which is further processed by Cas proteins to small protective CRISPR RNAs (called crRNAs) (Brouns et al., 2008; Pougach et al., 2010; Djordjevic et al., 2012). crRNAs are responsible for recognition and, together with Cas proteins, inactivation of invading foreign genetic elements (Brouns et al., 2008; Al-Attar et al., 2011). Cas proteins also take part in CRISPR adaptation, which is a process in which new spacers from viral genomes are inserted in CRISPR array. Figure 1 shows a schematic gene diagram for Type I-E CRISPR-Cas from *E. coli*, (Mojica and Diez-Villasenor, 2010; Patterson et al., 2017), which we consider in this paper. The *cas* genes and the CRISPR array are transcribed from separate promoters, which are located inside of the intergenic regions here denoted by IGLB and L (the leader sequence), respectively (see Figure 1; Pougach et al., 2010; Pul et al., 2010).

Promoters for *cas* operon and the CRISPR array are repressed in Type I-E CRISPR-Cas in *E. coli* (Pougach et al., 2010; Pul et al., 2010; Westra et al., 2010), which makes this system silent under standard conditions. Consequently, to generate crRNAs that can protect the bacterial cell, CRISPR-Cas has to be activated. Thus, to understand the system function it is crucial to understand the main features that control dynamics of CRISPR-Cas activation (Mojica and Diez-Villasenor, 2010; Richter et al., 2012; Patterson et al., 2017). However, approaching this problem experimentally is complicated due to the following:

- It requires direct experimental observation of *in vivo* dynamics of molecular species (proteins or RNA) in a cell (see e.g., Morozova et al., 2015).
- The signaling which leads to system induction is currently unclear (Ratner et al., 2015; Patterson et al., 2017), e.g., even a viral infection, an obvious trigger, is not sufficient to activate the system.
- To understand the roles of the key system features in its response/dynamics these features would have to be perturbed, which may require extensive reengineering of the system.

A complementary approach is to use mathematical/biophysical modeling to assess how different features of CRISPR-Cas expression affect system dynamics. Moreover, *in silico* analysis allows one to study alternative system architectures, and/or to perturb the natural system (see e.g., Rodic et al., 2017), which in turn allows understanding the role of its key regulatory features.

Experimental research has led to a consistent picture of the main CRISPR-Cas regulatory features in closely related *E. coli* and *Salmonella enterica* (Pul et al., 2010; Westra et al., 2010; Medina-Aparicio et al., 2011). Under standard conditions, promoters for both CRISPR array and *cas* genes are repressed by global regulators (H-NS and LRP). Repression by these regulators is highly cooperative, as their binding is nucleated at certain position, and then extends along the DNA through cooperative interactions between repressor molecules (Bouffartigues et al., 2007). Additional regulators, such as CRP, may also be involved in the repression of *cas* operon (Yang et al., 2014). While the exact signaling mechanism remains unclear, this repression must be relieved upon appropriate external signal (e.g., envelope stress that may signal bacteriophage invasion), through the action of transcription activators (LexA, LeuO, and BaeR-S are likely involved) (Richter et al., 2012; Patterson et al., 2017). In particular, for Type I-E CRISPR-Cas in *E. coli*, it was shown that cooperative repression by H-NS can be relieved by elevated amount of LeuO (Pul et al., 2010; Westra et al., 2010). Thus, highly cooperative repression, which is abolished by transcription activators, emerges as a major feature of CRISPR-Cas transcription control in *E. coli* and its relatives.

**Figure 2:**
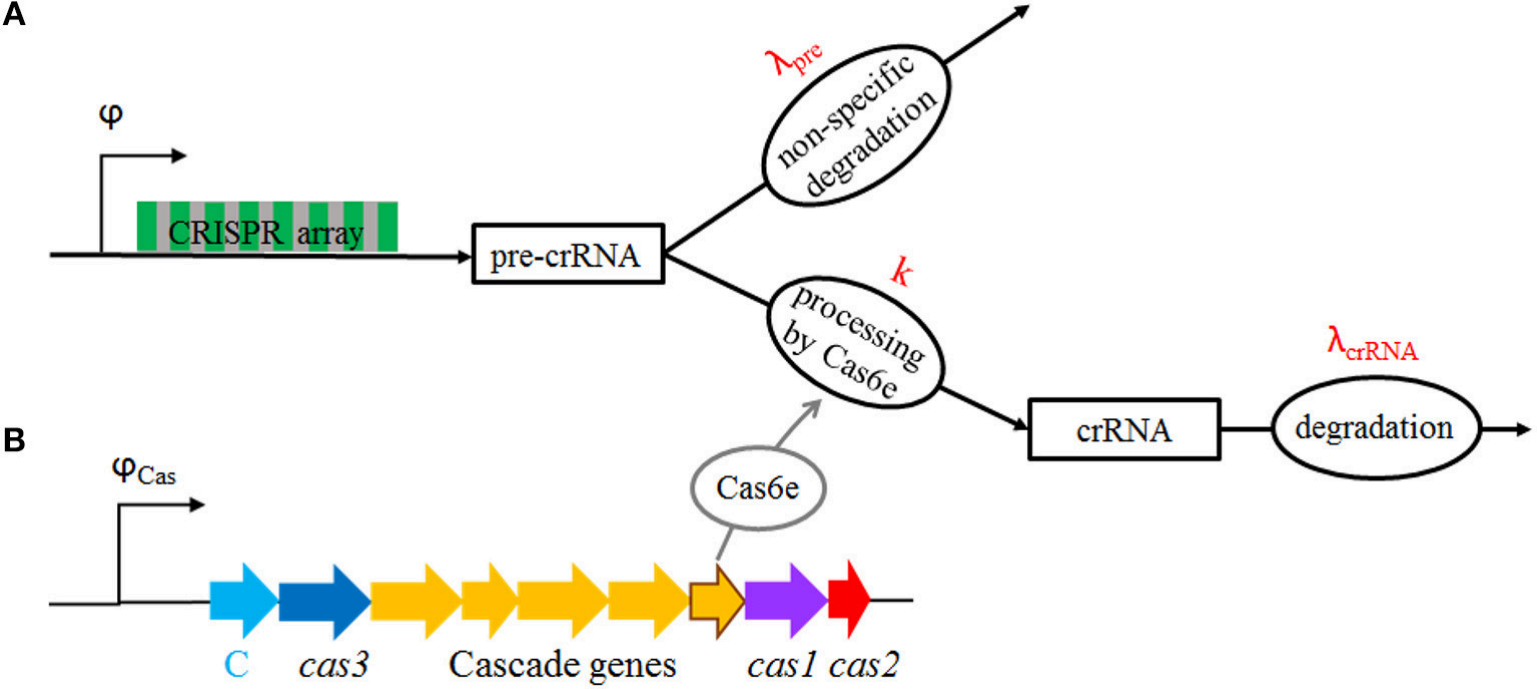
**(A)** A scheme of CRISPR transcript processing: CRISPR array is transcribed (i.e., pre-crRNA is generated) with rate φ, and the transcript is either (non-specifically) degraded with rate λ_*pre*_, or processed to crRNAs by Cas6e with rate *k*; individual crRNAs are then degraded with rate λ_*crRNA*_ (Djordjevic et al., 2012). **(B)** The proposed model system for CRISPR-Cas activation: *cas* genes (including *cas6e*, whose product processes pre-crRNA to crRNA), and the transcription factor (C), are transcribed from φ_*Cas*_ promoter. To reproduce the same qualitative features of transcription regulation as in a native CRISPR-Cas system (cooperative regulation), φ_*Cas*_ is put under control of C protein, in the same manner as in a well-studied AhdI R-M system (Bogdanova et al., 2008). The system is induced when the plasmid expressing *cas* genes and C protein enters a bacterial cell, as indicated in Figure 3. Gradual expression of *cas* genes, leading to Cas6e protein synthesis (gray oval), then increases *k* (this is indicated by the full arrow in the figure), which in turn results in crRNA generation.

Another crucial mechanism in CRISPR-Cas expression is pre-crRNA transcript processing (Brouns et al., 2008; Pougach et al., 2010). Experiments in *E. coli*, reported that overexpression of Cas6e (which is responsible for pre-crRNA processing) generates highly abundant crRNAs from pre-crRNA which is present at low abundance (Pougach et al., 2010). We previously showed that a simple quantitative model—whose relevant kinetic scheme is shown in Figure 2A—explains this observation (Djordjevic et al., 2012), so that a small decrease in pre-crRNA abundance leads to a much larger (around two orders of magnitude) increase in crRNA abundance. Interestingly, the main mechanism responsible for this strong amplification is fast non-specific degradation of pre-crRNA (see Figure 2) by unidentified nuclease(s). In particular, when cas genes expression increases, processing of pre-crRNA by Cas6e is favored and diverts the entire pre-crRNA molecule away from the path of non-specific degradation. Therefore, the fast non-specific degradation of pre-crRNA should be considered as a second major regulatory feature of CRISPR-Cas expression.

The modeling described in Djordjevic et al. (2012) took into account only the transcript processing step, i.e., it was assumed that there is an infinitely abrupt (stepwise) increase of pre-crRNA to crRNA processing rate, and pre-crRNA generation rate. This is, however, a clear idealization of the induction mechanism, as transcription regulation of *cas* genes and CRISPR array promoters is neglected. That is, in reality, pre-crRNA processing rate can be increased only gradually, as it takes time to synthesize the needed Cas proteins. The rate of Cas proteins synthesis is in turn directly related to the transcription control of the *cas* gene promoter in the IGLB region (see Figure 1). Similarly, the rate by which pre-crRNA is synthesized is determined by the transcription control of the CRISPR array promoter (L region).

Consequently, a more realistic model of CRISPR-Cas expression dynamics has to take into account both the regulation of CRISPR array and Cas protein synthesis, and CRISPR transcript processing. However, a major obstacle in achieving such model is that signaling which leads to the system induction, and detailed mechanism of CRISPR-Cas transcription regulation, is still unclear. We here propose a model system for CRISPR-Cas induction by assuming that activation of crRNA production is put under transcriptional control exhibited in a restriction-modification (R-M) immune system (Pingoud et al., 2014). As argued below, such model system would have qualitative features of transcription regulation expected for a CRISPR-Cas, and will keep the same transcript processing mechanism as that described for native system. On the other hand, this model system allows bypassing the currently unknown signaling that leads to CRISPR-Cas activation, and can be readily analyzed *in silico*, since transcription regulation of a well-studied R-M system (AhdI, see Bogdanova et al., 2008)—for which we previously showed that it can be reliably modeled (see below)—is used as a proxy for transcription regulation of CRISPR-Cas system.

Through this approach, we expect to:

- Obtain quantitatively more realistic model of CRISPR-Cas induction dynamics, in which the transcription regulation, i.e., the gradual synthesis of relevant enzymes and transcription regulators is explicitly taken into account.
- Qualitatively understand the main features of CRISPR-Cas induction, in particular the roles of cooperative transcription regulation, and of fast non-specific degradation of pre-crRNA.
- Propose an experimental setup for CRISPR-Cas induction that mimics the main qualitative features of the native system.

The setup of the model will be explicitly considered in the next subsection.

## Results

### *In silico* experiment setup

#### The model system

We start from a CRISPR transcript processing scheme, which is shown in Figure 2. According to this scheme, pre-crRNA is generated with rate φ, and subsequently either non-specifically degraded (due to activity of an unspecified nuclease) with rate λ_*pre*_, or is processed by Cas6e to crRNAs with rate *k*. crRNAs are subsequently degraded with rate λ_*crRNA*_. All the parameters in the scheme are experimentally determined in (Djordjevic et al., 2012) (for Type I-E CRISPR-Cas in *E. coli*) and explicitly stated in Methods. In particular, the main feature of the transcript processing is a large (non-specific) pre-crRNA degradation rate (with λ_*pre*_ ~ 1 1/min), which is much larger than crRNA degradation rate (with λ_*crRNA*_ ~ 1/100 1/min). In the experiments, crRNA production is artificially activated, by overexpressing Cas6e from a plasmid, which increases pre-crRNA processing rate (*k*) for between one and two orders of magnitude (between 10λ_*pre*_ and 100λ_*pre*_). While the repression of the *cas* promoter in IGLB region (see Figure 1) is very strong, with very small amount of Cas6e synthesized when the system is uninduced, the repression of the CRISPR array promoter is significantly weaker, with rather strong basal rate of pre-crRNA generation (φ ~ 10 1/min) (Pougach et al., 2010; Pul et al., 2010; Westra et al., 2010; Djordjevic et al., 2012).

As indicated in the Introduction, we previously modeled the transcript processing mechanism (Djordjevic et al., 2012), where we took that *k* is increased abruptly, i.e., as a step function at *t* = 0. This neglects the transcription regulation of *cas* and CRISPR array promoters. Such abrupt increase of *k* will provide a baseline for our predictions, which will now take into account that Cas6e (the enzyme which processes pre-crRNA to crRNA) is synthesized gradually. While in the experiments crRNA generation is activated by overexpressing Cas6e from a plasmid (see e.g., Pougach et al., 2010), it is likely that in the native system the expression of CRISPR array is activated as well (Pul et al., 2010). Consequently, we will also take into account a gradual synthesis of the regulator [in our case, a C-protein (Tao et al., 1991; Bogdanova et al., 2008)], which can activate CRISPR array transcription by increasing the basal rate φ to a higher value.

**Figure 3:**
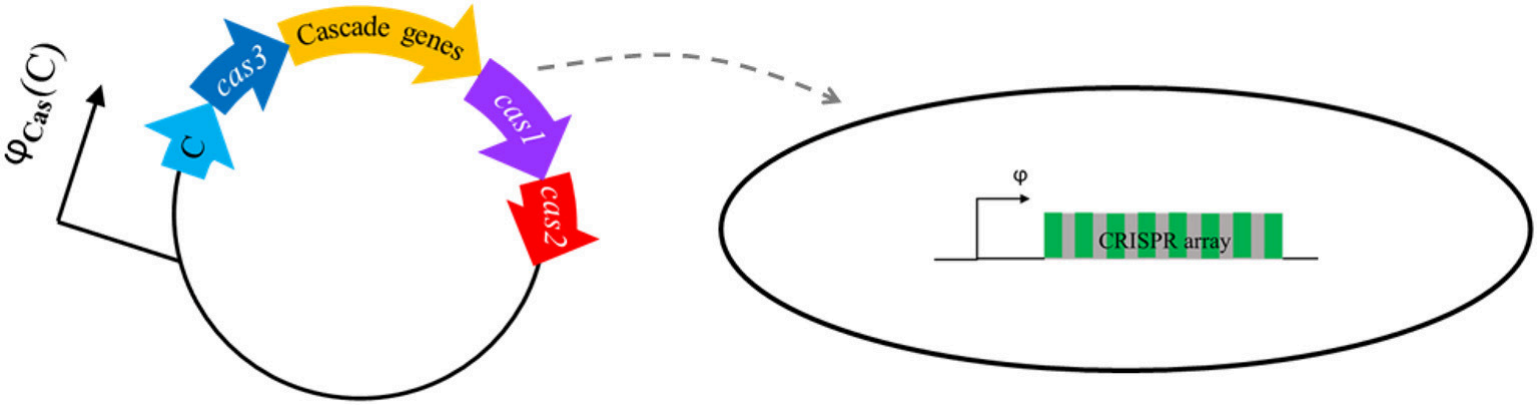
A scheme of the basic setup: pre-crRNA is transcribed in a cell from CRISPR array with rate φ. Transcription of *cas* genes occurs from a plasmid, which enters the cell, inducing crRNA generation. A transcription regulator (C) is transcribed together with *cas* genes, regulating transcription of the φ_*Cas*_ promoter in a same qualitative manner as exhibited in native CRISPR-Cas system. To achieve this, C protein controls transcription of φ_*Cas*_ promoter in the same way as in a well-studied R-M system. This leads to a gradual synthesis of Cas6e and C protein, leading to system activation, as schematically shown in Figure 2B.

To include transcription regulation of the *cas* promoter, i.e., the gradual synthesis of Cas6e and C transcriptional regulator, we here propose the model system whose setup is schematically shown in Figures 2, 3. This setup includes a CRISPR array which is expressed from a promoter with basal transcription activity φ (Figure 3). The second component is a vector (plasmid, virus) which expresses *cas* genes and the control protein C that are jointly transcribed from a promoter with transcription activity φ_*Cas*_. While Cas3 is not directly relevant for the problem considered here (dynamics of crRNA generation), as it does not take part in crRNA biogenesis, it is necessary for CRISPR interference (Hille and Charpentier, 2016). We therefore include it in the setup to allow expression of all *cas* genes, i.e. to have a fully functional CRISPR-Cas system.

As detailed below, φ_*Cas*_ is regulated by C. To mimic the qualitative features of transcription regulation in native CRISPR-Cas system, we employ the transcription regulation found in some R-M systems, as explained in the next subsection. The system is activated when the vector enters a bacterial cell lacking its own *cas* genes, which leads to a gradual synthesis of Cas proteins (including Cas6e), therefore increasing the processing rate *k*, which in turn leads to crRNA generation (see Figure 2B—the full arrow) by pre-crRNA processing. Gradual increase of pre-crRNA generation rate can be also considered through this model, through activation of CRISPR array promoter by gradually synthesized C.

Note that the setup above, where *cas* genes are introduced in a cell on a vector, allows bypassing the unknown signaling step in CRISPR-Cas induction. That is, the vector entering the cell marks the start of the system activation (setting zero time in the dynamics simulations), and mimics the signaling which starts synthesis of the transcription activator. Therefore, the key regulatory features which characterize the downstream steps (CRISPR array transcription and transcript processing) can be studied both *in silico* (which will be done here), and also potentially experimentally. In terms of experimental implementation, introducing *cas* genes in a cell on a virus also allows synchronizing the cell population, which is an approach previously implemented to visualize R-M protein kinetics (Mruk and Blumenthal, 2008).

#### Putting CRISPR-Cas under transcription control of an R-M system

As discussed above, *cas* promoter will be put under transcription control exhibited by R-M systems. Below, the main elements necessary for modeling the system transcription regulation are introduced.

R-M systems are often mobile, and can spread from one bacterial host to the other (Mruk and Kobayashi, 2013). When a plasmid carrying R-M system genes enters a naive bacterial host, the host genome is initially unmethylated, and can consequently be cut by the restriction enzyme. It is, therefore, evident that expression of the restriction enzyme and methyltransferase must be tightly regulated in order to ensure that bacterial genome is protected by the methyltransferase (“antidote”), before it is cut by the restriction enzyme. This tight regulation is often achieved through a dedicated control (C) proteins (Tao et al., 1991; Vijesurier et al., 2000).

We here concentrate on the AhdI R-M system, whose transcription control by C protein has been well-studied (Bogdanova et al., 2008). The activation of AhdI by C protein is reminiscent of CRISPR-Cas activation, as strong cooperative interactions are involved in both cases. In particular, C proteins bound at promoter-proximal and promoter-distal operators interact with high binding cooperativity, so that configuration in which only one operator is occupied cannot be observed in the absence of RNA polymerase (RNAP). At lower C protein concentrations, RNAP can outcompete C protein bound at promoter-proximal operator, leading to transcriptionally active configuration (Bogdanova et al., 2009). Moreover, another feature exhibited in AhdI transcription control, i.e., autoregulation by C protein, is also likely found in CRISPR-Cas transcription regulation. That is, LeuO that activates CRISPR-Cas expression (Westra et al., 2010) also regulates its own transcription. In particular, similarly to transcription regulation of *cas* genes, *leuO* is repressed by H-NS, while this repression is abolished by LeuO (Chen et al., 2001). At high concentrations, C protein is bound at both promoter-proximal and promoter-distal position, leading to the promoter repression—see **Figure 5** in (Bogdanova et al., 2009) and the scheme of the transcription configurations shown in Figure 5 (framed in the figure). Negative autoregulation is also exhibited by LeuO, as it inhibits transcription activation of its gene by BglJ-RcsB (Stratmann et al., 2012). Therefore, putting *cas* genes under transcription control found in AhdI mimics the main qualitative features of CRISPR-Cas transcription regulation, namely, gradual synthesis of Cas proteins, cooperativity in transcription regulation, and putative autoregulation.

Another advantage of this setup is that we previously showed that biophysical modeling can be used to:(i) explain *in vitro* measurements of the *wild type* and mutant R-M system transcription control (Bogdanova et al., 2008), (ii) explain *in vivo* measurements of the system dynamics (Morozova et al., 2015), (iii) effectively perturb the main R-M system features and relate these perturbations with the system dynamics (Rodic et al., 2017). Consequently, transcription control of a well-studied AhdI R-M system, whose transcription regulation can be reliably modeled (Bogdanova et al., 2008), will serve as a proxy for the transcription control of a much less understood CRISPR-Cas system.

#### *In silico* analysis of the main system features

The baseline for our predictions will be provided by a model in which the increase of pre-crRNA to crRNA processing rate *k* is infinitely abrupt—we will call this the baseline model. Comparing the baseline model with predictions that take into account the system transcription regulation (as schematically shown in Figures 2, 3), allows analyzing how gradual synthesis of Cas6e affects kinetics of crRNA generation.

While in the native CRISPR-Cas both *cas* genes and CRISPR array promoters are repressed by global regulators, the repression of *cas* genes was found to be much stronger (Pul et al., 2010; Westra et al., 2010)—consequently, when the system is (experimentally) artificially induced, this is commonly done by expressing only *cas* genes (Pougach et al., 2010; Semenova et al., 2016; Musharova et al., 2017). However, in the native system, it is likely that expression of both CRISPR array and *cas* genes is activated when the appropriate induction signal(s) is received (Pul et al., 2010). We will therefore investigate the system dynamics when only *cas* genes are activated (i.e., only pre-crRNA processing rate is gradually increased), and when *cas* genes and CRISPR array promoter transcription are jointly (and gradually) increased. Consequently, in both of the models introduced below (constitutive and cooperative), we will consider two options. First, when only transcription of *cas* genes is activated, while transcription activity of CRISPR array remains constant. Second, we will consider the case when the transcription activity of CRISPR array is increased as well.

**Figure 4:**
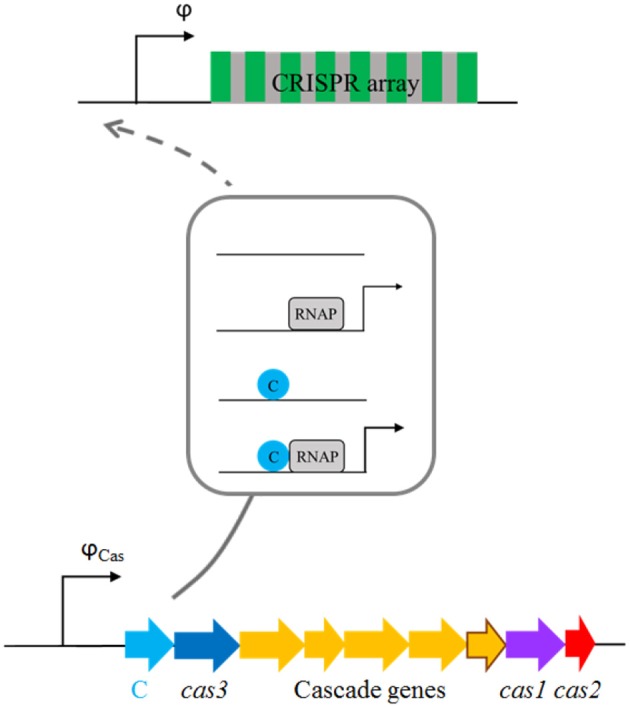
Transcription regulation of *cas* and CRISPR array promoters in the *constitutive model*. *C* and *cas* genes are transcribed from a constitutive promoter of constant strength φ_*cas*_. The CRISPR array promoter is either considered constitutive, with constant transcription activity (φ), or is regulated by C protein (indicated by the dashed arrow), where a scheme corresponding to this regulation is framed. The scheme shows possible configurations of CRISPR array promoter, where activation of CRISPR array transcription is achieved in the simplest manner, through the binding of a single C protein which acts as a transcription activator to the CRISPR array promoter. Transcriptionally active configurations are denoted by arrows, with thicker arrow indicating larger transcription activity.

**Figure 5:**
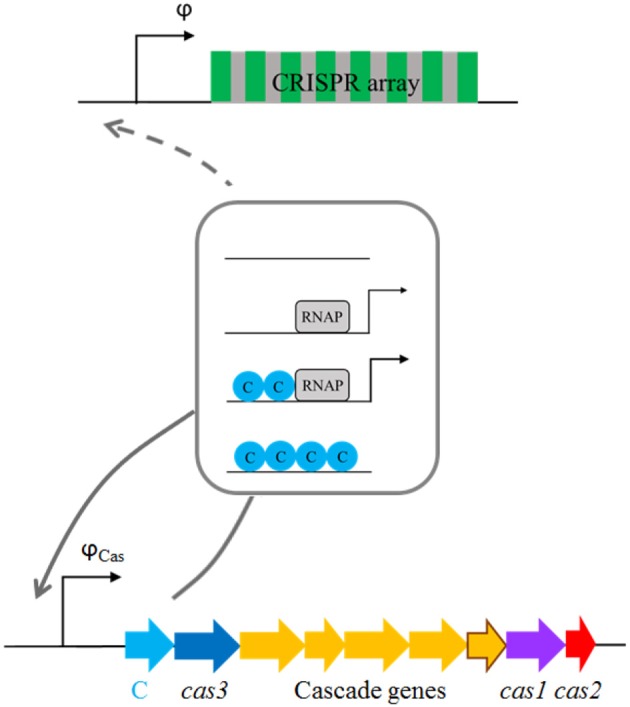
Transcription regulation of *cas* and CRISPR array promoters in the *cooperative model*. The framed scheme shows promoter configurations, where transcription regulation is exhibited in the same manner as for *Ahd*I system, through cooperative interactions. Arrows in the scheme denote transcriptionally active configurations, with thicker arrow indicating larger promoter transcription activity. The full gray arrow indicates that *cas* promoter is regulated as described by the scheme, with the same parameters as in *Ahd*I R-M system (Pougach et al., 2010). The dashed arrow indicates that the same transcription regulation is also exhibited for CRISPR array promoter, in the case when its transcription activity φ is not assumed constant.

We further introduce two models of *cas* gene and CRISPR array transcription regulation:

- *The constitutive model* (Figure 4). In this model *cas* genes are expressed from a constitutive promoter, so that they are transcribed with the constant rate once the plasmid enters a cell. In the case when we consider that the system is activated by only increasing pre-crRNA processing rate, the transcription activity φ is kept constant. When CRISPR array transcription rate is increased as well, increasing φ is exhibited in the simplest manner, by binding of a single C protein activator. Note that, in accordance with its name, no cooperativity is exhibited for transcription regulation described by this model.
- *The cooperative model* (Figure 5). In this model, C protein regulates the transcription of *cas* genes, and its own transcription, in the same manner as in AhdI R-M system. As noted above, such transcription regulation is characterized by strong cooperative interactions. CRISPR array transcription rate is either kept constant, or in the case when it is increased, we take that it is exhibited in the same way as for *cas* promoter transcription (the dashed arrow in Figure 5).

Studying of the two models allows one to assess how the cooperative transcription regulation (which also characterizes the native CRISPR-Cas system) compares to the activation in which no cooperativity is exhibited, and therefore allows us to assess the role of this key system feature. Also, considering the two models when φ is first kept constant, and then increased together with *k*, allows assessing significance of CRISPR array transcription control. To allow a direct comparison of models dynamics, the overall strength of φ_*Cas*_ is adjusted so that the same value of maximal pre-crRNA processing rate is achieved. Similarly, when the transcription rate of CRISPR array is increased, the interaction parameters are adjusted so that the same equilibrium increase of φ is achieved in both models (see Methods).

### Modeling results

#### Kinetics of pre-crRNA and crRNA production

We first consider the situation in which crRNA generation is activated by expressing Cas proteins, such that the processing rate *k* is gradually increased, while the CRISPR array transcription activity remains constant. In this case, we compare the system dynamics for: (i) *baseline model*, in which the processing rate *k* is increased as a step function, which corresponds to the limit of infinitely fast system induction, (ii) constitutive model (see Figure 4), and (iii) cooperative model (see Figure 5).

In constitutive and cooperative models, the gradual synthesis of Cas6e leads to gradual change of transcript processing rate *k* (k^*^ is a processing constant):

(1) $$\begin{array}{l} k\left(t\right)=[Cas6e]\left(t\right)\cdot k^{*} \end{array}$$

**Figure 6:**
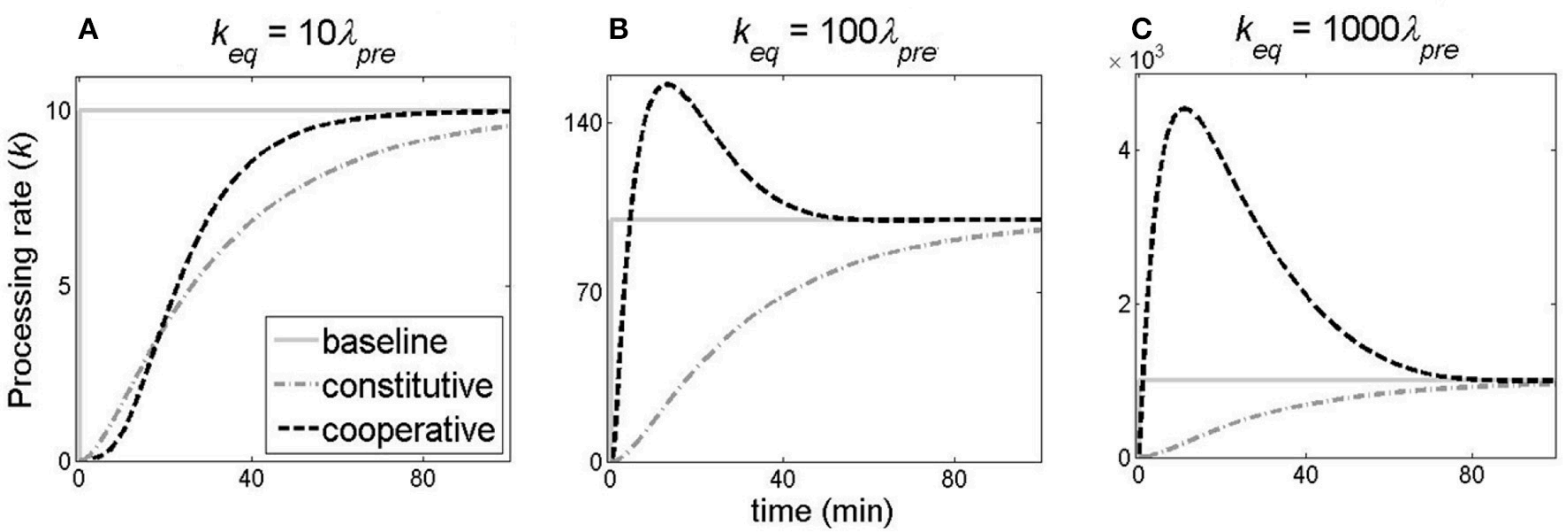
Comparing the dynamics of the pre-crRNA processing rate change. The change of the processing rate *k* with time is shown for: the baseline model (solid gray curve), the constitutive model (dash-dotted gray curve) and the cooperative model (dashed black curve). **(A–C)** correspond to different *k*_*eq*_ values (*k*_*eq*_ = 10λ_*pre*_, 100λ_*pre*_, 1,000λ_*pre*_, respectively). CRISPR transcription activity is constant (10 1/min).

Figure 6 illustrates how the processing rate (*k*) changes with time, when the baseline, constitutive, and cooperative models of *cas* gene expression are assumed. For the constitutive model (the dash-dotted curve), the processing rate uniformly increases and reaches an equilibrium value, for all values of *k*_*eq*_ considered in three panels of Figure 6. On the other hand, for cooperative model (the dashed curve) and at higher values of *k*_*eq*_ (Figures 6B,C), we see a rapid increase of *k* at initial times, followed by a fast return to the equilibrium value due to repression at higher C protein concentrations.

**Figure 7:**
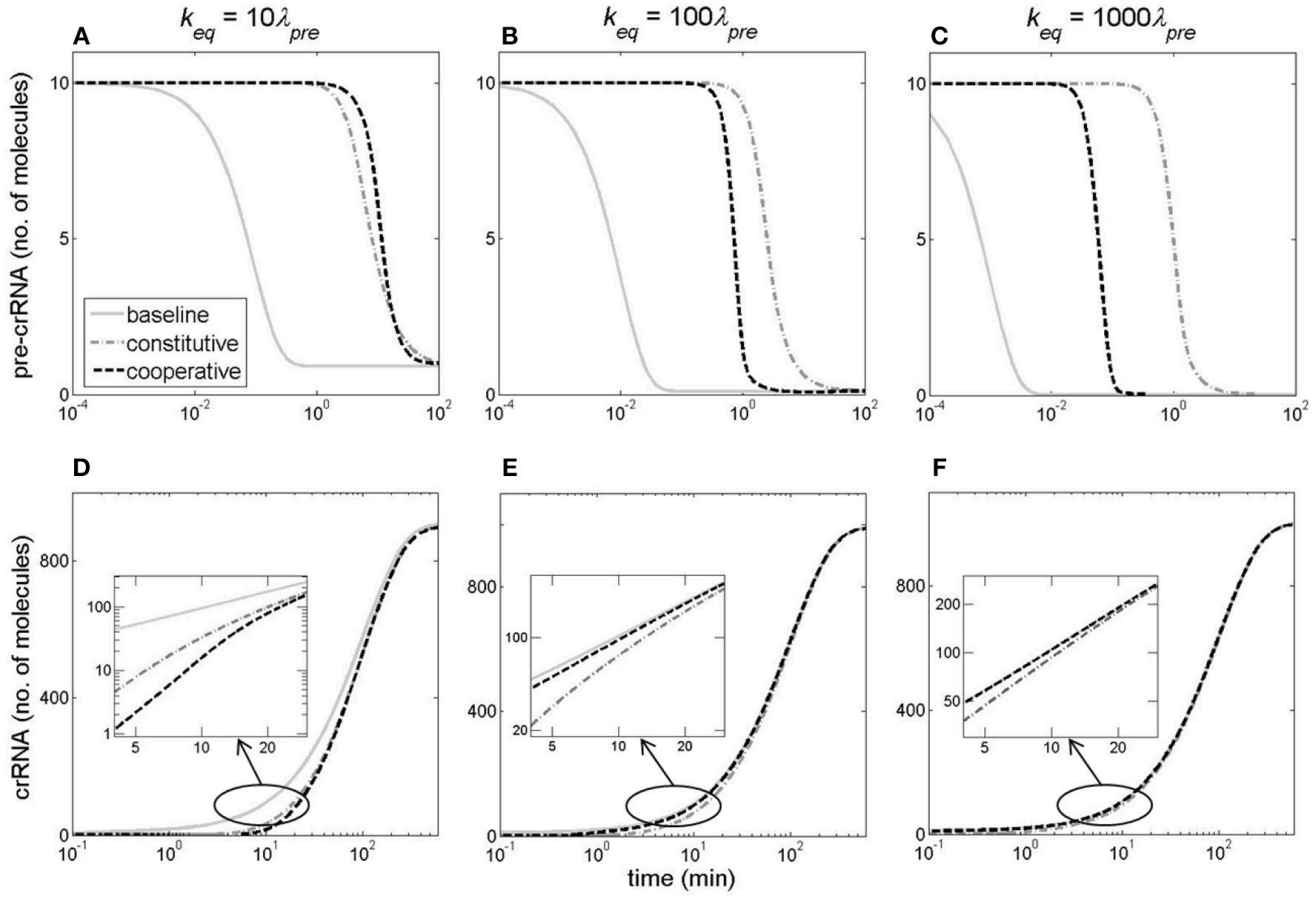
Kinetics of pre-crRNA and crRNA generation. The columns correspond to *k*_*eq*_ values of 10λ_*pre*_ **(A,D)**, 100λ_*pre*_ **(B,E)**, and 1,000λ_*pre*_ **(C,F)**, which are reached through the baseline model (the gray solid curve), the constitutive model (the gray dash-dotted curve) or cooperative model (the black dashed curve). The upper **(A–C)** and the lower **(D–F)** rows correspond, respectively, to pre-crRNA and crRNA kinetics. CRISPR array promoter transcription activity is kept constant at 10 1/min.

In Figure 7, we address how different *k* dynamics (shown in Figure 6), affects pre-crRNA and crRNA generation. Specifically, φ is held constant at its initial value (10 1/min), while *k* changes according to the baseline, constitutive, or cooperative models until reaching the same equilibrium value of 10λ_*pre*_, 100λ_*pre*_, and 1,000λ_*pre*_ (left, central, and right columns of Figure 7, respectively). The model of abrupt Cas6e expression serves as a baseline for assessing the dynamics in the other two models (constitutive and cooperative), in which Cas6e is realistically (gradually) expressed.

In Figures 7A–D, we see that cooperative model leads to the steepest transition from ON to OFF state (in the case of pre-crRNA), and from OFF to ON state (in the case of crRNA). Furthermore, we can distinguish between two different regimes in Figure 7. At lower *k*_*eq*_ (left column in Figure 7), there is a noticeably slower accumulation of crRNA at early times in both cooperative and constitutive models compared to the baseline model of infinitely abrupt processing rate (*k*) increase (Figure 7D). On the other hand, at higher *k*_*eq*_ (*k*_*eq*_≥100 1/min, the central and right columns in Figure 7), the dynamics of crRNA accumulation for cooperative model becomes faster compared to constitutive model dynamics at early times, and approaches the limit of infinitely abrupt *k* increase (see the inserts in Figures 7E,F). The faster kinetics of crRNA increase in cooperative model is due to the fast increase of *k* at early times in this model (Figures 6B,C).

#### Effects of *cas* genes regulation

**Figure 8:**
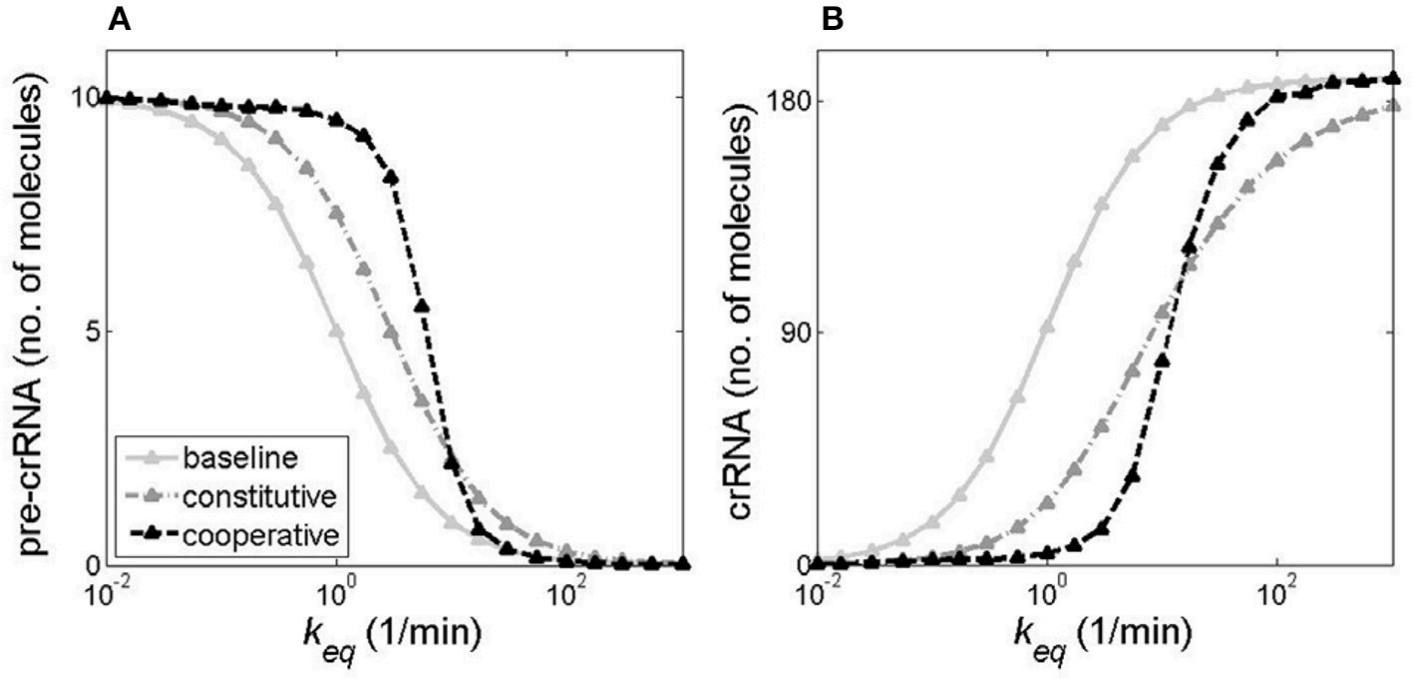
Pre-crRNA and crRNA amounts early post-induction for different models of *cas* gene transcription regulation. The figure shows **(A)** pre-crRNA and **(B)** crRNA amounts 20 min post-induction (i.e., 20 min after introduction of the vector expressing *cas* genes), as a function of the maximal (equilibrium) value of the transcript processing rate *k*. CRISPR promoter transcription activity is kept constant (φ = 10 1/min). The gray solid, the gray dash-dotted, and the black dashed curves correspond, respectively, to baseline, constitutive, and cooperative models of *cas* regulation.

From Figure 7, we observe that transcripts reach their steady-state levels quite late, i.e., >100 min post-induction. Such late time is, however, not relevant for cell response to phage infection, since infected *E. coli* lyse ~20 min post-infection, while shut-off of essential cell functions happens earlier (Kruger and Schroeder, 1981). Therefore, in Figure 8 we estimate pre-crRNA and crRNA levels for all three models at 20 min post-induction, as the maximal value of pre-crRNA processing rate *k*_*eq*_ is changed from very low to high values (>100λ_*pre*_, characteristic for artificial Cas6e induction), while keeping the level of CRISPR array transcription constant (φ = 10 1/min).

The following features emerge from Figure 8:

- A switch-like system behavior for both pre-crRNA and crRNA curves in the cooperative model, while the constitutive and baseline models yield much more gradual responses to changes in *k*_*eq*_. For crRNA, the cooperative model leads to a rapid transition from the OFF state (with essentially no crRNA generated at 20 min), to the ON state (with high abundance of crRNA), and reciprocal situation for pre-crRNA. Consequently, for small amounts of synthesized Cas6e (i.e., small *k*_*eq*_ values), which can be caused by leaks in *cas* promoter activity, the system remains in OFF state. On the other hand, once the system is activated when the processing rate (directly related to the amount of Cas6e available) reaches a certain threshold ($k_{eq}\underset{∽}{>}50$), a large amount of crRNA is generated at early times, which should allow protection from foreign DNA invasion. The significance of this behavior is considered in Discussion.
- An interesting cross-over behavior in the cooperative model, where at low *k*_*eq*_ values crRNA amounts are low, while at high *k*_*eq*_ values the synthesized crRNA amounts become larger than in the constitutive model, and approach the baseline model curve. Therefore, at high *k*-values (~100 1/min), which are encountered in experiments, (Pougach et al., 2010; Djordjevic et al., 2012) the model of cooperative *cas* gene expression leads to accumulation of protective crRNA amounts close to those achievable in the limit of infinitely abrupt *k* increase. Consequently, the high cooperativity in transcription regulation, characteristic for native CRISPR-Cas system regulation, leads to a highly efficient crRNA generation at high transcript processing rates.
- Sufficient crRNA levels are generated to protect host cell against bacteriophage infection, at early times post-induction, even at relatively low values of pre-crRNA processing rate. That is, *k*_*eq*_ somewhat larger than 11/min leads to ~10 crRNAs which already corresponds to the amount that negatively affects phage development (Pougach et al., 2010); moreover, a small additional *k*_*eq*_ increase leads to a large increase in generated crRNAs in the cooperative model, due to the rapid transition from OFF to ON state.
- A saturation in generated crRNA amounts at early times post-induction. That is, for *k*_*eq*_~100 1/min the amount of generated crRNAs at 20 min stops significantly increasing with further increase in *k*_*eq*_. This saturation can be relieved (leading to increase in the amount of generated crRNA), if CRISPR array transcription activity is increased, which is further analyzed below.

#### Perturbing pre-crRNA degradation rate

We next perturb the second key feature of CRISPR-Cas regulation—fast non-specific degradation of pre-crRNA. The consequence of pre-crRNA degradation rate λ_*pre*_ decrease at constant φ was next investigated for all three models. The decrease was followed at different *k*_*eq*_ values (i.e., at different levels of Cas6e activity), where φ is held constant.

**Figure 9:**
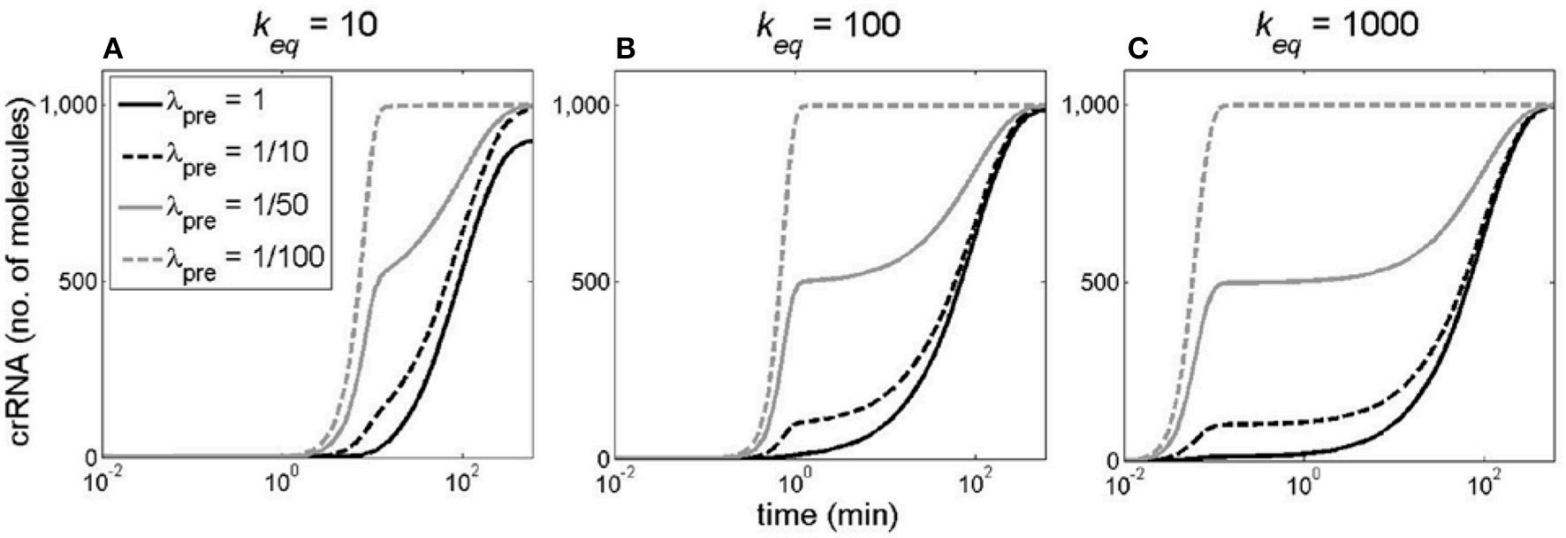
The effect of perturbing pre-crRNA degradation rate on the dynamics of crRNA generation. The pre-crRNA processing rate increases to its equilibrium value through the cooperative model, while φ is held constant (at 10 1/min). Different curves correspond to different λ_*pre*_ values: 1 (solid black), 1/10 (dashed black), 1/50 (solid gray), and 1/100 1/min (dashed gray). **(A–C)**, correspond to different *k*_*eq*_ values indicated at the top of each panel.

The effects of λ_*pre*_ decrease are similar for all three models, so in Figure 9 we show the results only for the cooperative model. For all *k*_*eq*_ values we see that abolishing the fast decay of pre-crRNA (decreasing λ_*pre*_), significantly decreases the time delay of the onset of crRNA generation. This effect is most pronounced at high *k*_*eq*_ values (Figure 9C). Also, perturbing the degradation rate deforms crRNA dynamics curve with respect to the standard Hill (sigmoidal) shape that is exhibited at high λ_*pre*_ such as λ_*pre*_ = 1/50. Furthermore, analogously to Figure 8, in Figure S1 (Supplementary Material), we show how crRNA amount at 20 min after induction depends on pre-crRNA degradation rate λ_*pre*_. One can clearly observe that as λ_*pre*_ decreases, the amount of generated crRNA early post-induction significantly increases, consistently with the decrease of the time delay of onset of crRNA generation observed in Figure 9.

#### Relieving crRNA production saturation by increasing pre-crRNA generation

In addition to *cas* genes, CRISPR array promoter is also repressed (though more weakly) by global transcription regulators (Pul et al., 2010; Westra et al., 2010). Consequently, crRNA generation can be also augmented by increasing CRISPR array transcription activity. Therefore, we next assess how joint increase of *k* (achieved by activating *cas* gene transcription) and φ (achieved by increasing CRISPR array transcription) affects generated crRNA amount 20 min post-induction for all three regulatory models.

**Figure 10:**
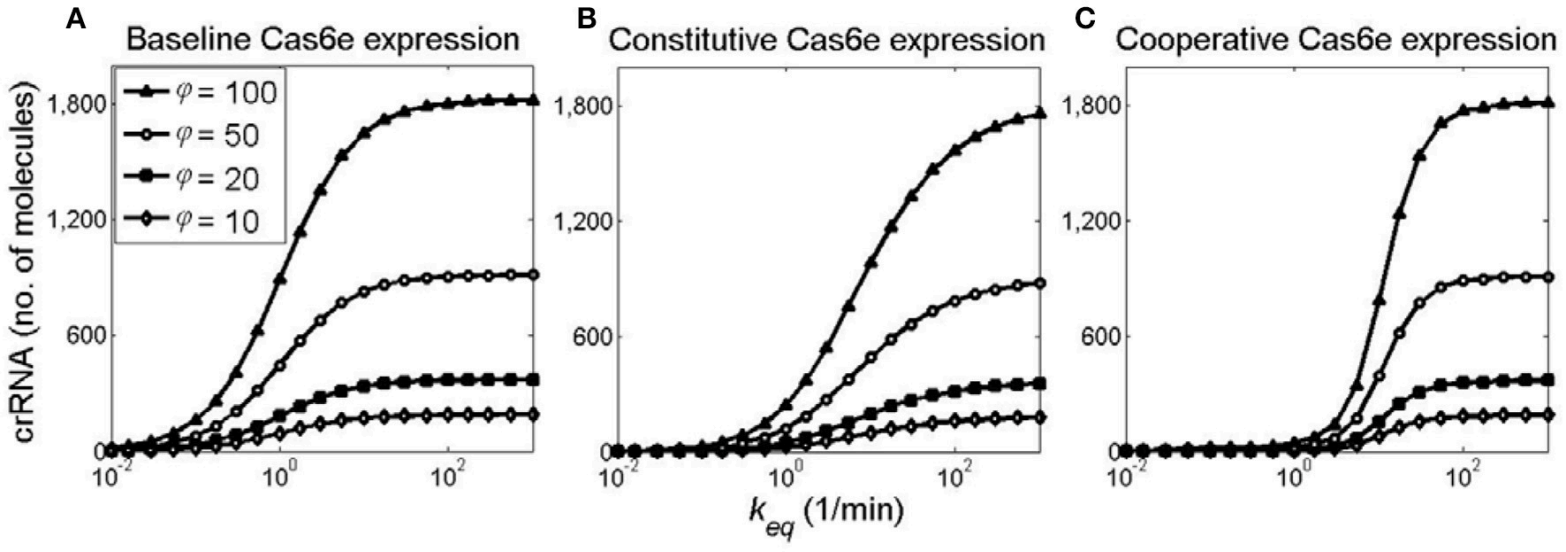
Relieving saturation in generated crRNA amounts through joint *k* and φ increase. crRNA amount as a function of *k*_*eq*_ at 20 min post-induction, obtained for **(A)** baseline, **(B)** constitutive, and **(C)** cooperative models. Curves marked with diamonds, filled squares, circles, and filled triangles, correspond, respectively, to φ of 10, 20, 50, and 100 1/min.

As can be seen from Figure 10, increasing φ robustly relieves crRNA saturation (see also discussion of Figure 8). Moreover, one can see that a relatively modest, factor of two increase of φ (from 10 1/min to 20 1/min) can abolish the need of a significant, order of magnitude, *k* increase to produce the same amount or crRNA. As above, we observe a switch-like behavior for the cooperative model (compare Figure 10C with Figures 10A,B), with cooperative model curves exhibiting the steepest transition from OFF to ON state for all φ values.

#### Regulation of CRISPR array transcription activity

We next consider how different models of regulation of CRISPR array transcription affect crRNA dynamics. For all three models, the transcription activity φ is increased by an order of magnitude (from φ = 10 1/min to φ = 100 1/min), for different *k*_*eq*_ values (*k*_*eq*_ = λ_*pre*_, 10λ_*pre*_, and 100λ_*pre*_), see Figure S2 (Supplementary Material). We obtain that the cooperative model leads to a more controlled (attenuated) pre-crRNA dynamics, which is due to the presence of repressing mechanism at high C protein amounts (see Figure S3). For crRNA dynamics, we observe that the cooperative model exhibits the steepest transition from OFF to ON state. Moreover, this model leads to the largest delay in crRNA generation. Consequently, in addition to pre-crRNA degradation rate, the cooperative transcription regulation also contributes to the delay between the activating signal and the onset of crRNA generation.

We previously (Figure 9) perturbed pre-crRNA degradation rate while keeping the transcription rate φ constant. Finally, we now also decrease λ_*pre*_ under the conditions when both *cas* genes and CRISPR array transcription is activated according to all three models (see Figure S4). The results are qualitatively similar to Figure 9 (where φ is constant), i.e., decreasing λ_*pre*_ diminishes the switch-like system response and/or decreases the time-delay in the onset of pre-crRNA generation.

## Discussion and summary

One of the most prominent problems in understanding CRISPR-Cas function is assessing dynamics of the system activation, i.e., understanding the roles of the key features of CRISPR-Cas regulation. Addressing this problem is complicated by the fact that exact conditions for system activation remain unclear. In fact, for Type I-E CRISPR-Cas system in *E. coli*, even bacteriophage infection itself is not sufficient to induce the system. We here proposed a synthetic setup which allows inducing CRISPR-Cas with qualitative features that correspond to native system regulation, while bypassing currently unclear conditions under which the system is activated. This setup involves putting *cas* genes and/or CRISPR array under transcription control found in a well-studied R-M system, which exhibits cooperative transcription regulation that is also characteristic of CRISPR-Cas regulation (Bouffartigues et al., 2007; Westra et al., 2010). A major advantage of the setup is that it can be readily experimentally implemented, e.g., by introducing *cas* genes and the regulator (C protein) in a cell on a virus. This would allow synchronizing the cell population, and experimentally observing the system dynamics, where such measurements could be directly compared with the predictions provided here. Another advantage is that major parameters in the setup have been inferred from experimental data, as both CRISPR transcript processing, and AhdI transcription regulation, have been experimentally well-studied (Bogdanova et al., 2008; Pougach et al., 2010; Djordjevic et al., 2012).

Consequently, this setup allows us to directly (*in silico*) address how the system regulation contributes to its dynamical response. In particular, previous experimental and computational work point to cooperative regulation of *cas* gene and CRISPR array transcription, and fast non-specific degradation of pre-crRNA, as two main system regulatory features (Pougach et al., 2010; Pul et al., 2010; Westra et al., 2010; Djordjevic et al., 2012). We therefore investigated two alternative regulatory architectures, one with constitutive, and the other with cooperative *cas* gene regulation. The dynamics corresponding to these two architectures was then compared with the baseline model, in which pre-crRNA processing rate is increased infinitely abruptly. We assessed the dynamics in the case when only *cas* genes are activated (i.e., only pre-crRNA processing rate is gradually increased), and when *cas* genes and CRISPR array promoter transcription is jointly increased. We focused on early system dynamics (within the first 20 min post-induction), as this period is most relevant for defending the cell against invading viruses. Finally, we also perturbed the high pre-crRNA non-specific degradation rate, under different system conditions described above, and assessed what effect such perturbation has on system dynamics.

The main result of the analysis is that the system regulation leads to a clear switch-like behavior, characterized by an initial delay of crRNA synthesis, followed by a steep transition from OFF to ON state. Unexpectedly, it is not only the cooperative transcription regulation, but also fast non-specific pre-crRNA degradation, which leads to such dynamics. That is, decreasing the high pre-crRNA degradation rate effectively abolishes the delay in crRNA generation, and deforms the crRNA kinetics from the standard sigmoidal (Hill) shape (Hill, 2013) typical for switch-like system response (Figure 9). Interestingly, we also found that, when pre-crRNA processing rate and CRISPR array transcription rate are jointly (and gradually) increased, as likely exhibited in the native system, the system is more robust to perturbations in the degradation rate (Figure S4).

The cooperative transcription regulation leads to an interesting cross-over behavior in the early system dynamics. At low pre-crRNA processing rates, cooperative regulation leads to much smaller crRNA amounts at early times compared to constitutive expression. On the other hand, at higher processing rates, there is a large increase in synthesized crRNA amounts, which approach the limit of infinitely abrupt system induction. Interestingly, when the system is artificially activated by overexpressing *cas* genes, pre-crRNA processing rates correspond to the regime of the highly enhanced crRNA production (Djordjevic et al., 2012). While the parameters of the native system induction are unclear, it is tempting to hypothesize that they may also reach this cross-over, allowing the system to generate crRNAs with the rate close to the limit of infinitely fast induction at times when they are needed.

The rapid transition of the system from OFF to ON state is straightforward to interpret in terms of its function in immune response. When a potential signal indicating infection is received by the cell, CRISPR-Cas has a very short time to generate sufficient crRNA amounts to protect the cell, as bacteriophages are typically highly efficient in shutting-down essential cell functions. Thus, there is a question whether enough crRNA can be generated in a model which accounts for gradual synthesis of proteins that process pre-crRNA and/or are responsible for gradual CRISPR array activation. We robustly obtained that enough crRNA can be generated at early times, even when the system is activated by only increasing the pre-crRNA processing rate. Moreover, a much smaller increase of the processing rate is needed to achieve certain crRNA amount, if CRISPR array transcription is activated as well. Therefore, these results may explain the relatively inefficient repression of CRISPR array promoter, since even a small increase of CRISPR array transcription rate efficiently increases generated crRNA amounts. In fact, the need to rapidly produce large amounts of crRNAs may be a major constraint on system dynamics.

In distinction to the rapid transition of the system from “OFF” to “ON” state, interpretation of the delay in crRNA generation, which comes as a model prediction, is less straightforward. One possibility is that such delay is related with primed adaptation in CRISPR-Cas, which relies on a pre-existing (priming) spacer that enables a biased uptake of new spacers—therefore serving to minimize infection by phage escape mutants that would otherwise evade the interference (Sternberg et al., 2016). In particular, it has been found that priming is facilitated by slow or delayed CRISPR interference, leading to a steady-state flux of substrates from which new spacers can be acquired (Kunne et al., 2016; Severinov et al., 2016; Musharova et al., 2017). Such delay in CRISPR interference can clearly be achieved by a delay in crRNA generation that is predicted in our work.

It has been proposed that Type I-E CRISPR-Cas in *E. coli* may have functions other than immunity. For example, it was found by bioinformatics analysis that the system is changing very slowly, in distinction to rapid diversification of CRISPR arrays in other species, indicating that the system is not taking an active role in defense against immediate viral threats (Touchon et al., 2011). In this respect, it may be useful to view the dynamical properties inferred here in a more general terms, namely of a capability of expressing a large number of molecules in a narrow time interval, with a specific time-delay with respect to reception of an external signal. It is clear that such highly efficient, and temporally specific response, may be highly desirable for multiple cellular functions. It would be very interesting to find out how functions of *E. coli* Type I-E CRISPR-Cas, yet to be discovered in the future, would fit within the dynamical properties inferred here.

## Methods

We start from a previously introduced model of CRISPR transcript processing by Cas proteins (Djordjevic et al., 2012). In this model (see Figure 2A), a short-living transcript [pre-crRNA] is synthesized with a promoter transcription activity φ, and further, either quickly degraded with a degradation rate λ_*pre*_, or processed (cut) into shorter, long-living RNAs [crRNA] with a processing rate *k*. Processed transcripts are degraded with a rate λ_*crRNA*_. In the equations below, we assume that the processing rate depends linearly on the substrate (pre-crRNA) amount, since the amount of pre-crRNA is small [ < 10 molecules per cell (Pougach et al., 2010)], so that the corresponding kinetic equations are:

(2) $$\begin{array}{l} \frac{d[\text{pre}-\text{crRNA}]}{dt}=\varphi -(\lambda_{\text{pre}}+k)\cdot [\text{pre}-\text{crRNA}] \end{array}$$

(3) $$\begin{array}{l} \frac{d[\text{crRNA}]}{dt}=k\cdot [\text{pre}-\text{crRNA}]-\lambda_{\text{crRNA}}\cdot [\text{crRNA}] \end{array}$$

The equations above are further solved deterministically, as both CRISPR array and *cas* genes are expressed from promoters with strong basal transcription. Furthermore, the small pre-crRNA amount is due to fast non-specific degradation, i.e., due to the transcript processing step. With respect to this, note that there is an access of enzyme (Cas6e) over substrate (pre-crRNA) (Djordjevic et al., 2012), so the equations describing the transcript processing are linear. Therefore, their deterministic solution accurately describes the mean of the stochastic simulations.

In the previous study (Djordjevic et al., 2012), we considered a model in which transcription regulation is neglected, so that *k* and φ increase in an idealized manner, i.e., infinitely abruptly. We now introduce models where the relevant enzymes and transcription regulators are synthesized in a realistic (i.e., gradual) manner. Specifically, *k* in Equation now explicitly depends on time, and is proportional to the enzyme (the processing protein, Cas6e) concentration, i.e., *k* = [*Cas*6*e*]·k^*^, where k^*^ is processing constant. We here consider that this processing rate *k* can change with time in the following ways:

1. Infinitely abruptly, from 0 to its equilibrium value, *k*_*eq*_ at *t* = 0, which we refer to as the baseline model.
2. Gradually, with [Cas6e](t), where Cas6e is expressed from a constitutive promoter (promoter with constant transcription activity), see Figure 4.
3. Also gradually with [Cas6e](t), where Cas6e is now expressed from an AhdI-like regulated promoter (see Figure 5).

As noted above, we either keep the CRISPR array transcription rate φ constant (which allows us investigating the dynamics in response to changing only pre-crRNA processing rate), or allow φ to change:

1. Infinitely abruptly (the baseline model), so that at *t* = 0 it increases from its starting value (10 1/min) to the equilibrium value.
2. Gradually, through the simplest activation mechanism, where a single C protein activates transcription from the CRISPR array promoter (the dashed arrow in Figure 4).
3. Also gradually with C(t), where the same transcription regulation as in AhdI RM system is exhibited (the dashed arrow in Figure 5).

In constructing Cas6e and CRISPR expression models, we refer to our existing model of AhdI restriction-modification (RM) system control (Bogdanova et al., 2008), which describes expression of the control protein (C) and the restriction endonuclease (R)—C and R are co-transcribed in AhdI RM system. We here use a thermodynamical model of CR operon transcription regulation, and a dynamical model of transcript and protein expression.

For *t* = 0 we take the moment when plasmid carrying C and *cas* genes enters the naïve host. Thus, all initial conditions are set to zero, except for [pre-*crRNA*](*t* = 0) = φ/λ_*pre*_ = 10(1/min)(Djordjevic et al., 2012), as extracted from the Equation in equilibrium. Note that while C and *cas* genes enter the cell on a plasmid, CRISPR array is expressed within the cell, with the transcription rate φ.

### Constitutive model of *cas* gene and CRISPR array expression

We assume that C and *cas* genes are co-transcribed from a constitutive (unregulated) *cas* promoter (see above and Figure 4). C and *cas* transcript and protein concentrations change with time:

(4) $$\begin{array}{l} \frac{d[c-cas](t)}{dt}=\varphi_{Cas}-\lambda_{Cas}\cdot [\text{c}-\text{cas}](t) \end{array}$$

(5) $$\begin{array}{l} \frac{dC(t)}{dt}=k_{C}\cdot [\text{c}-\text{cas}](t)-\lambda_{C}\cdot C(t) \end{array}$$

(6) $$\begin{array}{l} \frac{d[Cas6e](t)}{dt}=k_{Cas6e}\cdot [\text{c}-\text{cas}](t)-\lambda_{Cas6e}\cdot [\text{Cas6e}](t). \end{array}$$

Note that all the notation (including in the equation above), is introduced in Table 1. The first terms on the right-hand side represent transcript/protein synthesis by transcription/translation, while the second terms represent transcript/protein decay by degradation. The parameter values are as in AhdI RM system model (with Cas6e now replacing R in AhdI system), and are also provided in the table at the end of the methods. Since C and Cas6e protein degradation rates are taken to be the same, it follows:

(7) $$\begin{array}{l} {}[\text{Cas6e}](t)=\frac{k_{Cas6e}}{k_{C}}C(t), \end{array}$$

So that the differential equation for Cas6e dynamics can be omitted. We set the value of φ_*Cas*_ to one (see the next subsection) so that the equilibrium processing rate is the same for the constitutive and the cooperative models (see e.g., Figure 6), which allows a direct comparison of the dynamics in these two models. Consequently, we set k^*^ so that $k_{eq}={\left[Cas6e\right]}_{eq}\cdot k^{*}=10\left(1/\min\right)$. Regarding CRISPR array transcription φ, we keep it constant, in the case when we consider the system activation by overexpression of *cas* genes. In the case when we also consider activation of CRISPR transcription, we introduce a simple model of CRISPR expression regulation (the dashed arrow in Figure 4), where CRISPR promoter, apart from being unoccupied, can be found in the following three configurations, which are represented by the reactions shown below: (i) RNAP alone bound to the promoter (8), (ii) a C monomer alone bound to its binding site (9), and (iii) RNAP recruited by a C monomer bound to its binding site, acting as a transcription activator —note that these configurations correspond to the second, third and fourth line in the framed part of Figure 4, respectively.

(8) $$\begin{array}{l} DNA+RNAP\underset{K_{1A}}{⇄}RNAP-DNA \end{array}$$

(9) $$\begin{array}{l} DNA+C\underset{K_{2A}}{⇄}C-DNA \end{array}$$

(10) $$\begin{array}{l} C-DNA+RNAP\underset{K_{3A}}{⇄}C-DNA-RNAP \end{array}$$

The equilibrium dissociation constants of the above reactions are given by:

(11) $$\begin{array}{l} K_{1A}=\left[DNA\right]\left[RNAP\right]/\left[RNAP-DNA\right] \end{array}$$

(12) $$\begin{array}{l} K_{2A}=\left[DNA\right]\left[C\right]/\left[C-DNA\right] \end{array}$$

(13) $$\begin{array}{l} K_{3A}=\left[C-DNA\right]\left[RNAP\right]/\left[C-DNA-RNAP\right]. \end{array}$$

Using the Shea-Ackers based approach, i.e. assuming that the transcription activity is proportional to the equilibrium promoter occupancy by RNAP, we derive the expression for CRISPR promoter transcriptional activity:

(14) $$\begin{array}{l} \varphi =\gamma \frac{Z_{RNAP}+Z_{C-RNAP}}{1+Z_{RNAP}+Z_{C}+Z_{C-RNAP}} \end{array}$$

where γ is a proportionality constant, while configuration statistical weights correspond to: $Z_{RNAP}^{\;}=\left[RNAP-DNA\right]/\left[DNA\right]$ − RNAP alone bound to the promoter, *Z*_*C*_ = [*C*−*DNA*]/[*DNA*]–C monomer alone bound to its binding site, *Z*_*C*−*RNAP*_ = [*C*−*DNA*−*RNAP*]/[*DNA*] − RNAP recruited to the promoter by a bound C monomer. We can obtain φ dependence on C concentration:

(15) $$\begin{array}{l} \varphi \left(C\right)=\gamma \frac{d+def\left[C\right]}{1+d+e\left[C\right]+def\left[C\right]} \end{array}$$

If we introduce parameters expressed in terms of the equilibrium binding constants and RNAP concentration:

(16) $$\begin{array}{l} d=\left[RNAP\right]/K_{1A} \end{array}$$

(17) $$\begin{array}{l} e=1/K_{2A} \end{array}$$

(18) $$\begin{array}{l} f=K_{1A}/K_{3A}. \end{array}$$

To estimate the parameters, we use a condition:

(19) $$\begin{array}{l} \varphi (0)=10\frac{1}{\min} \end{array}$$

which corresponds to the value in Djordjevic et al. (2012), and:

(20) $$\begin{array}{l} \varphi (C_{eq})=100\frac{1}{\min} \end{array}$$

Another (evident) condition is that the fraction, which appears on the right-hand side of the Equation (15), has to be smaller than 1. By adjusting the parameters to satisfy the conditions (19) and (20), we obtain *d* < 1/9, which allows setting the values of d and γ. Further, we notice that *e* = 99/([*C*]_*eq*_·(*f* − 100)) and, having fixed the value of *f*, we can adjust *e* with respect to [C]_eq_.

**Table 1:** Notations used in model equations.

**Variables**	**Description**	
*φ_*Cas*_*	Transcription activity of *cas* promoter	
φ	Transcription activity of CRISPR promoter	
*[c-cas]*	Concentration of *cas* operon transcript	
*[pre-crRNA]*	Concentration of unprocessed CRISPR array transcript	
*[crRNA]*	Concentration of processed CRISPR array transcript	
*C*	Concentration of control protein	
*[Cas6e]*	Concentration of processing protein	
**KINETIC MODEL CONSTANTS**		
k^*^	CRISPR transcript processing constant	0.02
*k_*C*_*	Translation constant for control protein	0.60
*k_*Cas*6*e*_*	Translation constant for processing protein	3.00
*λ_*Cas*_*	Rate of *cas* transcript decay	0.20
*λ_*pre*_*	Rate of unprocessed CRISPR transcript decay	1.00
*λ_*crRNA*_*	Rate of processed CRISPR transcript decay	0.01
*λ_*C*_*	Rate of control protein decay	0.033
*λ_*Cas*6*e*_*	Rate of Cas6e processing protein decay	0.033
**TRANSCRIPTION REGULATION MODELS CONSTANTS**		
α	Proportionality constants	1.663
γ		110
α′		110
*a*	Constants which absorb the relevant equilibrium dissociation constants and RNA polymerase concentration	1.60 × 10^−1^
*p*		9.25 × 10^−1^
*q*		1.41 × 10^−5^
*d*		1.00 × 10^−1^
*e*		Adjusted
*f*		2.00 × 10^2^
*a*′		1.00 × 10^−1^
*p*′		Adjusted
*q*′		2.50 × 10^−5^
*K_*D*_*		6.50 × 10^2^

The unprocessed [pre-crRNA] and processed [crRNA] transcript amounts change with time according to the Equations (2) and (3), where φ is given by.

### Cooperative model of *cas* and CRISPR expression

As opposed to the constitutive *cas* operon expression, we here assume that the *cas* promoter is regulated by C as in the wild type AhdI RM system (Bogdanova et al., 2008), through cooperative interactions (see Figure 5). The following set of reactions describes the transcriptional regulation of the *cas* promoter by the C protein (note the promoter configurations shown in Figure 5):

(21) $$\begin{array}{l} C+C\underset{K_{1}}{⇄}D \end{array}$$

(22) $$\begin{array}{l} DNA+RNAP\underset{K_{2}}{⇄}RNAP-DNA \end{array}$$

(23) $$\begin{array}{l} D+DNA\underset{K_{3}}{⇄}D-DNA \end{array}$$

(24) $$\begin{array}{l} D-DNA+D\underset{K_{4}}{⇄}T-DNA \end{array}$$

(25) $$\begin{array}{l} D-DNA+RNAP\underset{K_{5}}{⇄}D-DNA-RNAP \end{array}$$

where C and D stand for C protein monomers and dimers, respectively.

The reactions (21)–(25) represent:

– (21) *C* monomers dimerization;
– (22) *RNAP* binding to the *cas* promoter forming *RNAP-DNA* complex;
– (23) *D* binding to the distal binding site forming *D-DNA* complex;
– (24) *D* recruitment to the proximal binding site forming *T-DNA* complex;
– (25) *RNAP* recruitment to the *cas* promoter forming *D-DNA-RNAP* complex.

In equilibrium the above reactions lead to the following equations of the equilibrium dissociation constants:

(26) $$\begin{array}{l} K_{1}=\frac{{\left[C\right]}^{2}}{\left[D\right]} \end{array}$$

(27) $$\begin{array}{l} K_{2}=\frac{\left[DNA][RNAP\right]}{\left[RNAP-DNA\right]} \end{array}$$

(28) $$\begin{array}{l} K_{3}=\frac{\left[D][DNA\right]}{\left[D-DNA\right]} \end{array}$$

(29) $$\begin{array}{l} K_{4}=\frac{\left[D][D-DNA\right]}{\left[T-DNA\right]} \end{array}$$

(30) $$\begin{array}{l} K_{5}=\frac{\left[RNAP][D-DNA\right]}{\left[D-DNA-RNAP\right]} \end{array}$$

Taking into account the aforementioned Shea-Ackers assumption we obtain:

(31) $$\begin{array}{l} \varphi_{Cas}=\alpha \frac{Z_{RNAP}+Z_{D-RNAP}}{1+Z_{RNAP}+Z_{D-RNAP}+Z_{T}}, \end{array}$$

α is a proportionality constant, *Z*_*RNAP*_ = [*RNAP*−*DNA*]/[*DNA*], *Z*_*D*−*RNAP*_ = [*D*−*DNA*−*RNAP*]/[*DNA*] and *Z*_*T*_ = [*T*−*DNA*]/[*DNA*] denote the statistical weights of only RNAP bound to the promoter, RNAP recruited to the promoter by a C dimer bound to the distal binding site, and a C tetramer repressing transcription, respectively.

By using Equations (26)–(30), the Equation (31) can be rewritten in terms of C monomer concentration (following the notation in Bogdanova et al., 2008; Rodic et al., 2017):

(32) $$\begin{array}{l} \varphi_{Cas}\left(C\right)=\alpha \frac{a+b{\left[C\right]}^{2}}{1+a+b{\left[C\right]}^{2}+c{\left[C\right]}^{4}} \end{array}$$

which can be expressed, by using the redefined parameters, in the following form:

(33) $$\begin{array}{l} \varphi_{Cas}\left(C\right)=\alpha \frac{a+ap{\left[C\right]}^{2}}{1+a+ap{\left[C\right]}^{2}+p^{2}q{\left[C\right]}^{4}}. \end{array}$$

We set α so that the equilibrium value of *cas* transcription activity corresponds to one (adapted from Bogdanova et al., 2008). Parameters *a, p*, and *q* depend on the equilibrium dissociation constants and RNAP concentration and are given by:

(34) $$\begin{array}{l} a=[RNAP]/K_{2} \end{array}$$

(35) $$\begin{array}{l} p=\frac{K_{2}}{K_{1}K_{3}K_{5}} \end{array}$$

(36) $$\begin{array}{l} q=\frac{1}{K_{1}^{2}K_{3}K_{4}p^{2}}=\frac{K_{3}K_{2}^{5}}{K_{2}^{2}K_{4}} \end{array}$$

While their values are deduced from the already determined a, b, and c, that correspond to the best fit to the AhdI experimentally measured transcription activity vs. C (Bogdanova et al., 2008).

Regarding the dynamics, note that C and Cas6e transcript and protein amounts change with time according to the Equations (4)–(6), where φ_*Cas*_ is given by.

Similarly as for the constitutive model, we keep φ constant, in the case when we consider inducing the system through increasing pre-crRNA processing rate. When we also consider regulation of CRISPR array transcription, we assume that CRISPR promoter is regulated by C in the same way as *cas* promoter. Thus, following the same procedure we obtain for the CRISPR promoter transcription activity:

(37) $$\begin{array}{l} \varphi =\alpha^{\prime }\frac{a^{\prime }+a^{\prime }p^{\prime }{\left[C\right]}^{2}}{1+a^{\prime }+a^{\prime }p^{\prime }{\left[C\right]}^{2}+p^{\prime 2}q^{\prime }{\left[C\right]}^{4}} \end{array}$$

where constants α′*, a*′*, p*′, and *q*′ are determined by imposing the same constraints on φ as above (-). Specifically, these constraints lead to the condition $a^{\prime }<\frac{1}{9}$, which allows setting parameters *a*′ and α′. Further, from Equation (20) we express *p*′ in terms of *q*′ and get $q^{\prime }<\frac{1}{400*99}$ (deduced from the real roots criterion of quadratic equation), based on which we set *q*′, and subsequently obtain the relation for adjusting *p*′ with respect to *k*_*eq*_ (i.e., *C*_*eq*_). Again, the unprocessed [pre-crRNA] and processed [crRNA] transcript amounts change with time according to the Equations (2) and (3), where φ is replaced with (37).

### Changing pre-crRNA processing rate

From Equation (1) we have that

(38) $$\begin{array}{l} k_{eq}={\left[Cas6e\right]}_{eq}\cdot k^{*}, \end{array}$$

where we adjust the equilibrium value of *k* in the constitutive and the cooperative case by varying the concentration of Cas6e in equilibrium. The equilibrium Cas6e concentration can be derived from the steady-state conditions for Equations and:

(39) $$\begin{array}{l} {\left[Cas6e\right]}_{eq}=\frac{k_{Cas6e}}{\lambda_{Cas}\lambda_{Cas6e}}\varphi_{Cas}(C_{eq}). \end{array}$$

In the model of constitutive C and Cas6e expression, the equilibrium concentration of Cas6e is adjusted through the change of φ_*Cas*_ (being constant with time). In the case of cooperative C and Cas6e expression, [*Cas6e*]_*eq*_ is adjusted through the change of α in Equation (33), i.e., through the change of overall *cas* promoter strength, taking into account that [C]_*eq*_ is proportional to [*Cas6e*]_*eq*_ according to (7).

### Joint change of *k* and φ

We here investigate how the joint change of *k* and φ, which corresponds to the joint increase of *cas6e* and CRISPR array gene expression, affects the dynamics of [pre-crRNA] and [crRNA] transcripts. We start from the baseline model of infinitely abrupt increase of *k* and φ. We then compare the baseline model to the more realistic case of constitutive and the cooperative models. We take φ change from the initial value of 10 1/min to 100 1/min in equilibrium, while *k*_*eq*_ takes on values λ_*pre*_, 10λ_*pre*_, and 100λ_*pre*_. Note that the change in *k*_*eq*_, implies joint change of φ_*Cas*_ in Equation (4) and *e* in Equation (15) in the constitutive case; in the cooperative case it implies joint change of α and *p* in Equation (33) and *p*′ in Equation (37), which ensures the same functional dependency φ*(t)*, for different values of *k*_*eq*_.

### Perturbing pre-crRNA degradation rate *λ_*pre*_*

The pre-crRNA degradation rate λ_*pre*_ is perturbed (decreased) in the following two cases:

- With the transcription rate φ (10 1/min) held constant. The equilibrium value of *k* is then adjusted by varying φ_*Cas*_ in the constitutive, and α in the cooperative model.
- When both φ and the processing rate *k* reach the equilibrium value (100 1/min) gradually, with the effect of the change assessed in all three models (baseline, constitutive and cooperative). *k*_*eq*_ reaches the value 100 1/min through the change of φ_*Cas*_ in the constitutive, and α and *p* in the cooperative model, while φ increases from φ(0) = 10 1/min to φ(*C*_*eq*_) = 1, 001/min through adjusting the parameters *e* in the constitutive, and *p*′ in the cooperative model.

Note that changing λ_*pre*_ affects the initial amount of pre-crRNA (which is an initial condition for the differential equations) according to the relation [*pre*−*crRNA*]_*eq*_(*t* = 0) = φ(*t* = 0)/λ_*pre*_ (see Equation 2), which follows from the steady-state condition for pre-crRNA when the system is not activated.

## Author contributions

All authors have given approval to the final version of the manuscript. MarD conceived and coordinated the work, with the help of KS and MagD. AR and BB performed calculations and the analysis. All the authors interpreted the results and contributed to writing the manuscript.

### Conflict of interest statement

The authors declare that the research was conducted in the absence of any commercial or financial relationships that could be construed as a potential conflict of interest.
