# Supplementary material for: Features of CRISPR-Cas Regulation Key to Highly Efficient and Temporally-Specific crRNA Production
Source: Front Microbiol. 2017 Nov 3;8:2139. doi: 10.3389/fmicb.2017.02139 (PMC5675862; doi:10.3389/fmicb.2017.02139)
Supplement: Supplementary file 1 [file Image1.PDF]

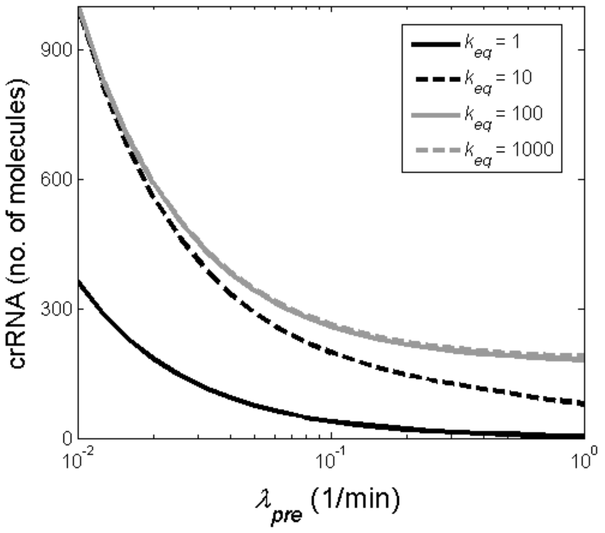

Figure S1. Effect of joint change in k_{eq} and λ_{pre} on the amount of crRNA reached at 20 min post-induction. Number of crRNA molecules 20 min post-induction is presented as a function of the pre-crRNA degradation rate λ_{pre}. Solid black, dashed black, solid grey and dashed grey curves correspond to increasing values of the equilibrium processing rate k_{eq}, as indicated in the figure legend.

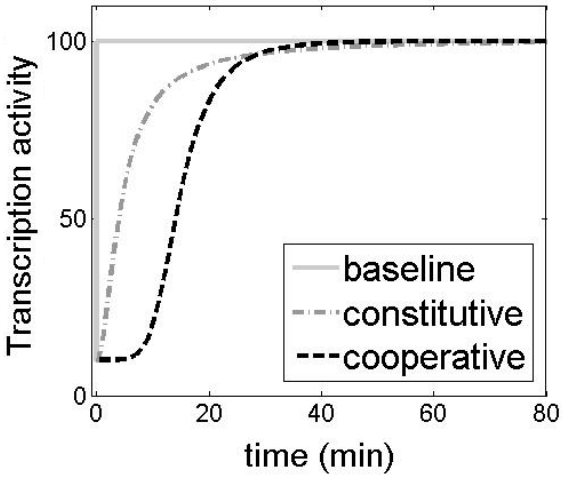

Figure S2. The change of CRISPR array transcription activity, for the baseline, constitutive, and cooperative models. CRISPR promoter activity (φ) reaches the equilibrium value of 100 1/min, which is an order of magnitude larger than its initial value (10 1/min). This increase is achieved by the baseline (the grey solid line), the constitutive (the grey dash-dotted line, see Fig. 4B) and the cooperative model (the black dashed line, see Fig. 5). The model parameters are chosen so that the dynamics of φ increase is independent of the maximal k_{eq} increase, i.e., φ dependence on time is the same for k_{eq} equaling λ_{pre}, $10\lambda_{pre}$ and $100\lambda_{pre}$ (see Methods).

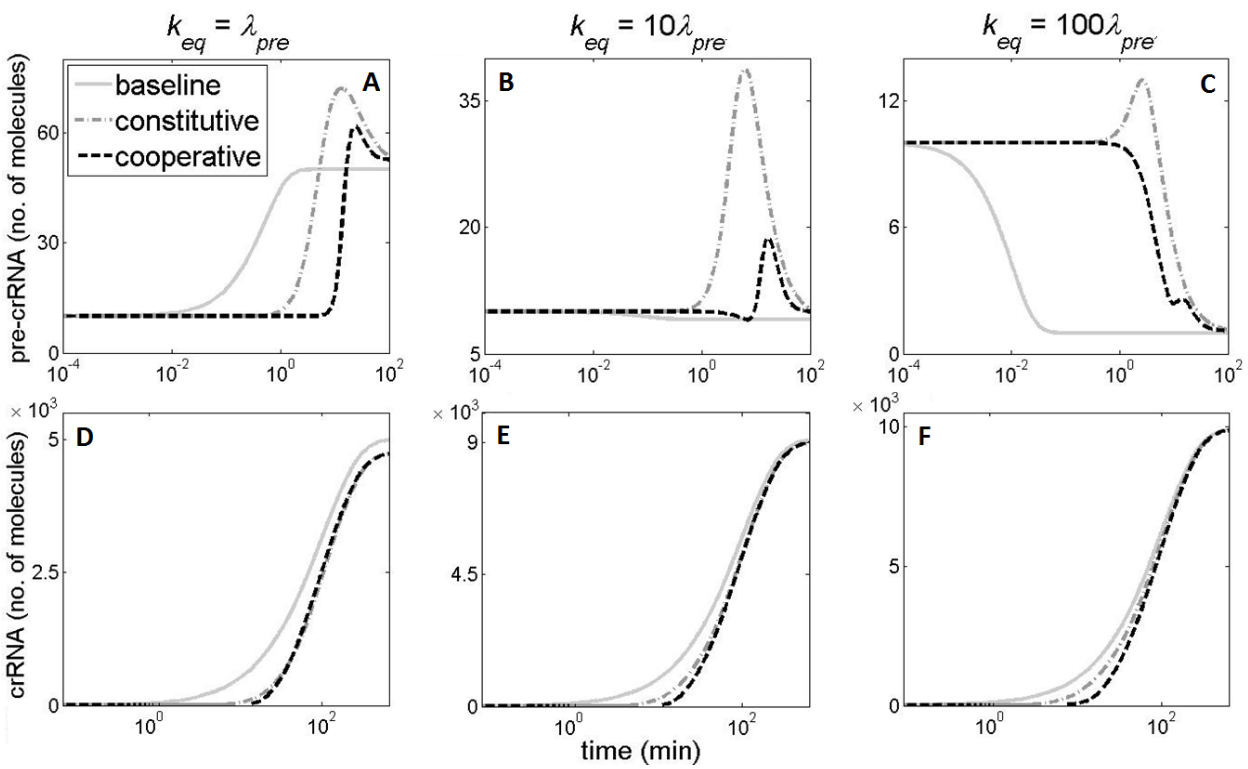

Figure S3. The effect of different CRISPR array transcription regulation on the dynamics of crRNA generation. Maximal CRISPR array promoter transcription activity is increased, from 10 1/min to 100 1/min. Grey solid, grey dash-dotted, and black dashed lines correspond, respectively, to the baseline, constitutive and cooperative models.. As in Fig. 7, k_{eq} is also increased, so that the first, the second, and the third columns correspond, respectively, to $k_{eq} = \lambda_{pre}$, $10\lambda_{pre}$, and $100\lambda_{pre}$. The first and the second row correspond to pre-crRNA and crRNA dynamics respectively.

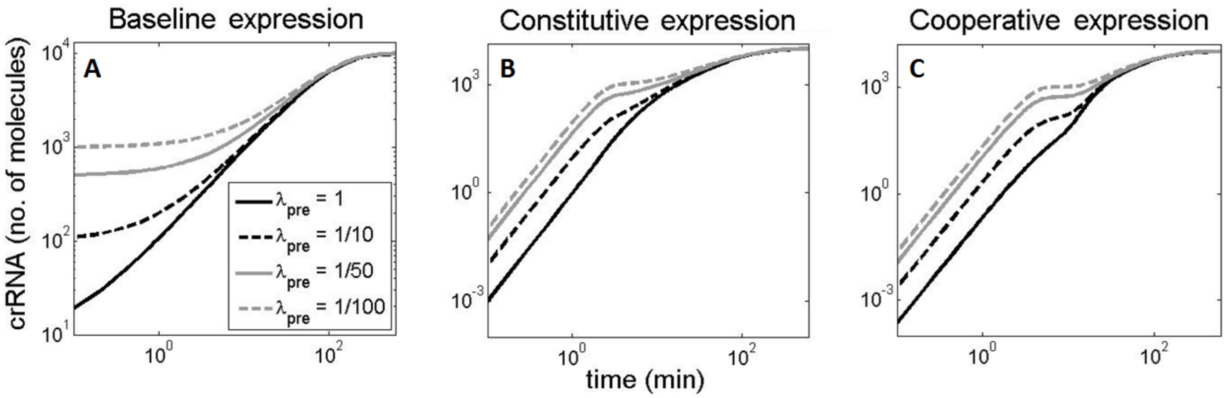

Figure S4. Perturbing pre-crRNA degradation rate for joint increase of k and φ. Dynamics of crRNA generation is obtained for baseline (**A**), constitutive (**B**) and cooperative (**C**) models of *cas* gene and CRISPR array expression. For each model, the processing rate k, and CRISPR array transcription rate reach the equilibrium value of 100 1/min, while λ_{pre} takes the following values: 1 (solid black curve), 1/10 (dashed black), 1/50 (solid grey) and 1/100 1/min (dashed grey).
